# Modelling timing and tempo of adrenarche in a prospective cohort study

**DOI:** 10.1371/journal.pone.0278948

**Published:** 2022-12-15

**Authors:** S. Ghazaleh Dashti, Lisa Mundy, Anne-Lise Goddings, Louise Canterford, Russell M. Viner, John B. Carlin, George Patton, Margarita Moreno-Betancur

**Affiliations:** 1 Clinical Epidemiology & Biostatistics Unit, Murdoch Children’s Research Institute, Melbourne, Victoria, Australia; 2 Department of Paediatrics, The University of Melbourne, Parkville, Victoria, Australia; 3 Centre for Adolescent Health, Murdoch Children’s Research Institute, Melbourne, Victoria, Australia; 4 Melbourne School of Psychological Sciences, The University of Melbourne, Melbourne, Victoria, Australia; 5 UCL Great Ormond Street Institute of Child Health, London, United Kingdom; Manisa City Hospital, TURKEY

## Abstract

To better understand how health risk processes are linked to adrenarche, measures of adrenarcheal timing and tempo are needed. Our objective was to describe and classify adrenal trajectories, in terms of timing and tempo, in a population of children transitioning to adolescence with repeated measurements of salivary dehydroepiandrosterone (DHEA), DHEA-sulphate, and testosterone. We analysed data from the Childhood to Adolescence Transition Study (CATS), a longitudinal study of 1239 participants, recruited at 8–9 years old and followed up annually. Saliva samples were assayed for adrenal hormones. Linear mixed-effect models with subject-specific random intercepts and slopes were used to model longitudinal hormone trajectories by sex and derive measures of adrenarcheal timing and tempo. The median values for all hormones were higher at each consecutive study wave for both sexes, and higher for females than males. For all hormones, between-individual variation in hormone levels at age 9 (timing) was moderately large and similar for females and males. Between-individual variation in hormone progression over time (tempo) was of moderate magnitude compared with the population average age-slope, which itself was small compared with overall hormone level at each age. This suggests that between-individual variation in tempo was less important for modelling hormone trajectories. Between-individual variation in timing was more important for determining relative adrenal hormonal level in childhood than tempo. This finding suggests that adrenal hormonal levels at age 8–9 years can be used to predict relative levels in early adolescence (up to 13 years).

## Introduction

Adrenarche, the reactivation of the hypothalamic-pituitary-adrenal (HPA) axis following a quiescent period in early childhood [1], is characterised by rising circulating levels of dehydroepiandrosterone (DHEA), dehydroepiandrosterone sulphate (DHEA-S), and testosterone [2, 3]. It is a process that varies between individuals not only in timing of onset but also potentially in duration and trajectories of hormonal change, as well as its relationship with gonadarche (the reactivation of the hypothalamic-pituitary-gonadal axis which also occurs during puberty [4]) [5].

Adrenarche has been posited as a sensitive period when exposure to different nutritional and social environments may alter patterns of growth and emotional development [6]. Early timing of adrenarche has been associated with heightened risk of metabolic problems, including obesity and polycystic ovary syndrome, mental health problems, and behavioural disorders [7, 8].

Further investigation of how health risk processes are linked to adrenarche requires disentangling adrenarche from gonadarche. It is necessary that these measures capture adrenarcheal timing of onset, tempo (speed of progress through adrenal development), and developmental trajectory [5] but currently, there are limited data available that explore these different aspects of adrenarche. Assessing tempo and timing of adrenarche phenotypically is difficult since the physical manifestations of adrenarche, including changes in body odour, axillary and pubic hair growth, and acne development, are frequently subtle until hormone increases have been present for some years [1]. Adrenal hormone measurements provide a measure of biochemical adrenarche, focussing on changing circulating hormone levels resulting from the HPA axis maturation, rather than phenotypic changes [5].

To differentiate between adrenarcheal timing and tempo requires longitudinal data, yet population-level data capturing longitudinal changes in adrenal hormonal markers are limited. Two studies have reported longitudinal DHEA-S hormonal trajectories. In a Chilean sample of 504 girls followed longitudinally, those with relatively higher levels of DHEA-S than their peers at age 7 also had higher DHEA-S and testosterone levels at the onset of breast development, with higher DHEA-S (but not testosterone) levels persisting one year after menarche [9]. A Danish study analysed longitudinal DHEA-S levels over 5 years in 179 children aged between 5.9 and 17 years at their first measurement and found that hormone levels increased with age until 13 years in both males and females [10], in keeping with the findings of other published cross-sectional studies (e.g., references [11–14]). None of these studies has addressed the question of adrenarcheal tempo.

The present study uses longitudinal data from the population-based Childhood to Adolescence Transition Study (CATS) [15], including repeated measures of salivary DHEA, DHEA-S, and testosterone across multiple time-points in children aged 8–9 years at their first measurement, to describe and classify individual hormone trajectories in terms of variations in adrenarcheal timing and tempo relative to the population averages. For this purpose, we use linear mixed-effects models with subject-specific random intercepts and slopes, a method that enables efficient estimation of the parts of the variance in longitudinal data that are attributable to within- and between-individual variation, yielding individual-level measures of variation relative to peers.

## Methods

Study data were drawn from the first four waves of CATS. The full study design is reported elsewhere [15]. A total of 1,239 Year 3 (8–9 years) children from primary schools in metropolitan Melbourne, Australia were recruited with written informed consent from parents/ guardians. The study was approved by the Royal Children’s Hospital Human Research Ethics Committee (#31089). The Victorian Department of Education and Training and Catholic Education in Melbourne granted permission to recruit through their schools.

Participants were followed annually for four years. At each wave, children completed a brief questionnaire, and their height, weight, and waist circumference were measured. At waves 1, 3 and 4 children were asked to provide a saliva sample, collected in the classroom setting using the passive drool method (see reference [15]). Most samples (83%) were collected between 9am-10am, when hormonal levels were likely to peak. At wave 2, children who had not provided a wave 1 saliva sample were invited to provide a sample and the time of saliva collection was not set. Children unable to provide 1 ml saliva were invited to provide a second sample which was collected in a small group setting. Students unable to attend the school session (4.5% of the sample) were provided with a home saliva collection kit to take an early morning sample. All components of data collection were voluntary.

### Saliva hormone measurement

Within 6 months of collection, saliva samples were allocated to batches (waves 1, 3, 4) and assayed or assayed as a single batch (wave 2). Before testing, thawed samples were weighed to approximate the volume of saliva collected (1g ~ 1ml), which was divided by the duration of saliva flow (min) to calculate the flow rate. Samples were then centrifuged at 3000 RPM at 4°C for 15 minutes. All samples were assayed in duplicate, using highly sensitive salivary enzyme immunoassay kits (Salimetrics, State College, PA), and the average hormone concentration was used in the analyses. For DHEA, the assay range was from 10.2 to 1,000 pg/mL; the average intraassay coefficient of variation (CV) was 9.9% and the average interassay CV was 16.5%. For DHEA-S, the assay range was from 62.9 to 15,300 pg/mL; average intraassay and interassay CVs were 8.3% and 9.1%, respectively. Following the manufacturer’s instructions, samples with DHEA-S values greater than 15,300 pg/mL were diluted with DHEA-S diluent, retested, and the newly obtained values were multiplied by the dilution factor. For testosterone, the assay range was 6.1 to 600 pg/mL, with average intraassay and interassay CVs 7.8% and 13.2%, respectively. Other assay specifications including analytical and functional sensitivity, and cross-reactivity data for each assay are included in S1 Table.

### Statistical analysis

#### Descriptive statistics

All analyses were stratified by sex. For each wave, age and salivary collection time were described using the mean and standard deviation (SD). Hormone measures were highly skewed and summarised as median and interquartile range. These variables were (natural) log-transformed for subsequent analyses.

#### Imputing values below the lowest detectable value

For each hormone, using the values measured above the lowest detectable value per the assay ranges, the distribution of the log-transformed values was approximated by a normal distribution, which was reasonable upon visual inspection, and the mean and SD of this distribution were estimated. The value that divided by two the area of this distribution below the log of the lowest detectable value was estimated, so that undetectable values had equal probability of lying above or below the estimated value. Undetectable values were imputed with that value (5.4 pg/ml for testosterone, 8.3 pg/ml for DHEA, 49.6 pg/ml for DHEA-S) on the log scale (S1 Table).

#### Linear mixed-effect models for saliva hormones

We used the mixed-effects approach to model the longitudinal data, which can be viewed as a compromise between using each individual’s noisy short sequence of observations to estimate their trajectory and assuming that all individuals follow the same “fixed” trajectory with only random measurement error [16]. In addition, the modelling framework allows irregular data, with varying numbers and times of observations for each individual, meaning that all the data available can be used in the analyses, and yields unbiased estimates under the “missing at random” assumption when hormone measurements are not available for all individuals across all time points [17].

For each hormone, linear mixed-effects models were fitted to the longitudinal hormone measurements including subject-specific random intercepts and age-slopes that represent individual measures of deviation from population trajectories in terms of the mean level at the age where the model is centered and rate of change, respectively. Models also included a batch-specific random effect to correct for batch effects. Fixed effects were specified for age centred at 9 years old, time at saliva collection centred at 9am (henceforth simply referred to as time), and an interaction between age and time. Specifically, hormone levels were modelled as increasing linearly with age and with time, with inclusion of an age×time interaction enabling the effect of diurnal variation to change as age increased (see S1 File for details). For (log) DHEA-S, the log-transformed flow rate was additionally included in all models with a fixed effect, because it is expected that the DHEA-S concentration (pg/ml) will fall as salivary flow (ml/min) increases [18]. Models were fitted using the standard method of restricted maximum likelihood [16]. The variance partitioning coefficient (VPC), which quantifies the proportion of the total variance in hormone measurements due to between-individual variation (versus within-individual and between-batch variation) among measurements at a given age was estimated from the variances in intercepts, slopes, random error, and the covariance between intercepts and slopes, estimated from the mixed-effects models [19].

#### Classification of individuals based on subject-specific hormone levels and rates of progression relative to population averages

For each hormone, estimates of the subject-specific random intercepts (denoted by $\hat{b_{0i}}$ for individual *i*) were taken as the measure for adrenarche timing. Individuals were grouped into three hormone classes (low-, mid-, or high-level) based on how their random intercept ($\hat{b_{0i}}$) compared with the tertiles of the distribution of *b*_0*i*_, estimated as *N* $\left(0,{\hat{\sigma }}_{b_{0}}^{2}\right)$, where ${\hat{\sigma }}_{b_{0}}^{2}$ is the random intercept variance estimated from the fitted linear mixed-effect model. Thus, for *q*_0*L*_ and *q*_0*U*_ representing the lower and upper tertiles of the estimated distribution, individual *i* would belong to:

Class L1 (low-level) if $\hat{b_{0i}}\leq q_{0L}$
Class L2 (mid-level) if $q_{0L}<\hat{b_{0i}}\leq q_{0U}$
Class L3 (high-level) if $tq_{0U}<\hat{b_{0i}}$

For each hormone, subject-specific random slope estimates (denoted by $\hat{b_{1i}}$ for individual *i*) were taken as the measure for tempo. Individuals were grouped into two classes (slow or fast rate) defined by whether their $\hat{b_{1i}}$ were above or below zero, which is the median of the assumed distribution of *b*_1*i*_ in the fitted linear mixed-effect models, estimated as $N\left(0,{\hat{\sigma }}_{b_{1}}^{2}\right)$. Thus, individual *i* belongs to:

Class R1 (slow rate) if $\hat{b_{1i}}\leq 0$
Class R2 (fast rate) if $\hat{b_{1i}}>0$

These classifications were combined to define the following six classes:

L1/R1: low-level, slow rate
L2/R1: mid-level, slow rate
L3/R1: high-level, slow rate
L1/R2: low-level, fast rate
L2/R2: mid-level, fast rate
L3/R2: high-level, fast rate

#### Missing data

The linear mixed-effect model used all available hormone measurements, even if an individual missed a wave, an analysis that yields unbiased model parameter estimates under the “missing at random” assumption when considering missingness in the hormone levels only [17]. However, participants with missing measurements across all waves were excluded, as well as those with missing values for any variables included in the analysis model (Table 1). For testosterone, measurements >300 pg/ml were treated as missing and excluded as they were apparent outliers and impeded the mixed-effect model convergence.

**Table 1:** Descriptive statistics for age at saliva collection, time of saliva collection, saliva flow rate, and hormone measurements at waves 1 to 4 for females (n = 667) and males (n = 572).

	Wave 1		Wave 2^a^		Wave 3		Wave 4	
	Females	Males	Females	Males	Females	Males	Females	Males
Provided saliva sample, n (%)	632 (94.8)	535 (93.5)	15 (2.2)	22 (3.8)	566 (84.9)	496 (86.7)	541 (81.1)	474 (82.9)
Sample collected at school, n (%)^b^	632 (100)	535 (100)	15 (100)	21 (95.5)	522 (92.2)	461 (92.9)	501 (92.6)	445 (93.9)
Age at saliva collection, years								
Mean (SD)	8.97 (0.36)	9.00 (0.41)	9.95 (0.33)	10.02 (0.27)	10.88 (0.37)	10.93 (0.40)	11.85 (0.37)	11.89 (0.40)
missing values, n (%)^c^	0	0	0	0	1 (0.1)	0	1 (0.1)	0
Time of saliva samples collection, hours								
Mean (SD)	9:51 (19 min)	9:51 (18 min)	11:34 (121 min)	12:04 (137 min)	9:30 (45 min)	9:29 (39 min)	9:21 (25 min)	9:21 (43 min)
missing values, n (%)^c^	0	0	1 (0.1)	1 (0.2)	3 (0.4)	7 (1.2)	4 (0.6)	13 (2.3)
**Saliva hormones** ^d^								
DHEA, pg/ml^e^								
<lowest detectable value, n (%)	106 (16.8)	148 (27.7)	1 (6.7)	2 (9.1)	20 (3.5)	47 (9.5)	9 (1.7)	37 (7.8)
Median [interquartile range]	34.01 [19.79, 57.41]	31.42 [17.21, 47.23]	44.33 [30.19, 55.21]	38.02 [24.80, 61.89]	74.76 [43.76, 109.85]	45.02 [26.38, 75.10]	75.05 [45.33, 119.67]	54.41 [31.31, 88.59]
missing values, n (%)^c^	1 (0.2)	1 (0.2)	0	0	0	0	2 (0.3)	1 (0.2)
DHEA-S, pg/ml^f^								
<lowest detectable value, n (%)	12 (1.9)	16 (3.1)	0 (0.0)	1 (4.5)	1 (0.2)	1 (0.2)	4 (0.8)	3 (0.6)
Median [interquartile range]	691.80 [355.17, 1332.21]	659.34 [311.66, 1294.57]	848.60 [583.54, 1080.46]	983.63 [738.45, 2217.05]	1742.30 [890.49, 3084.10]	1679.16 [815.61, 2926.13]	1788.76 [981.67, 3111.14]	1986.39 [1033.66, 3866.79]
missing values, n (%)^c^,^g^	12 (1.8)	11 (2.0)	1 (0.1)	1 (0.2)	8 (1.2)	7 (1.2)	21 (3.2)	21 (3.7)
Testosterone, pg/ml^g^								
<lowest detectable value, n (%)	21 (3.3)	29 (5.5)	0 (0.0)	0 (0.0)	3 (0.5)	0 (0.0)	2 (0.4)	1 (0.2)
Median [interquartile range]	20.97 [14.83, 28.47]	19.47 [13.39, 25.90]	22.36 [20.29, 29.16]	27.06 [21.49, 32.74]	39.01 [29.19, 52.44]	31.95 [23.06, 43.60]	39.86 [30.41, 55.76]	35.30 [26.08, 49.29]
missing values, n (%)^c^, ^i^	1 (0.2)	6 (1.1)	0	0	1 (0.2)	3 (0.5)	4 (0.6)	5 (0.9)

^a^At wave 2, only children who had not provided a saliva sample at wave 1 were invited to provide a sample and the time of saliva samples collection was not fixed in the morning.

^b^Percent reported among those who provided saliva sample.

^c^Missing values are reported among those who provided saliva sample.

^d^Medians and interquartile ranges for hormone levels are reported among participants with detectable values.

^e^Range of sensitivity was 10.2–1000 pg/ml.

^f^Range of sensitivity was 62.9–15300 pg/ml.

^g^Missing values for DHEA-S measurement or for saliva flow rate.

^h^Range of sensitivity was 6.1-600pg/ml.

^i^Testosterone values ≥300 pg/m (1 female, 2 male) were apparent outliers and treated as missing.

#### Additional analyses

As a sensitivity analysis for the impact of diurnal variation on classifications, all the classifications were repeated after excluding measurements from samples taken at or after 11am. To assess the impact of salivary collection environment, analyses were repeated after excluding saliva samples not collected at school.

Minimal underlying data set is uploaded to Open Science Framework: https://osf.io/6jdur/.

## Results

**Fig 1:**
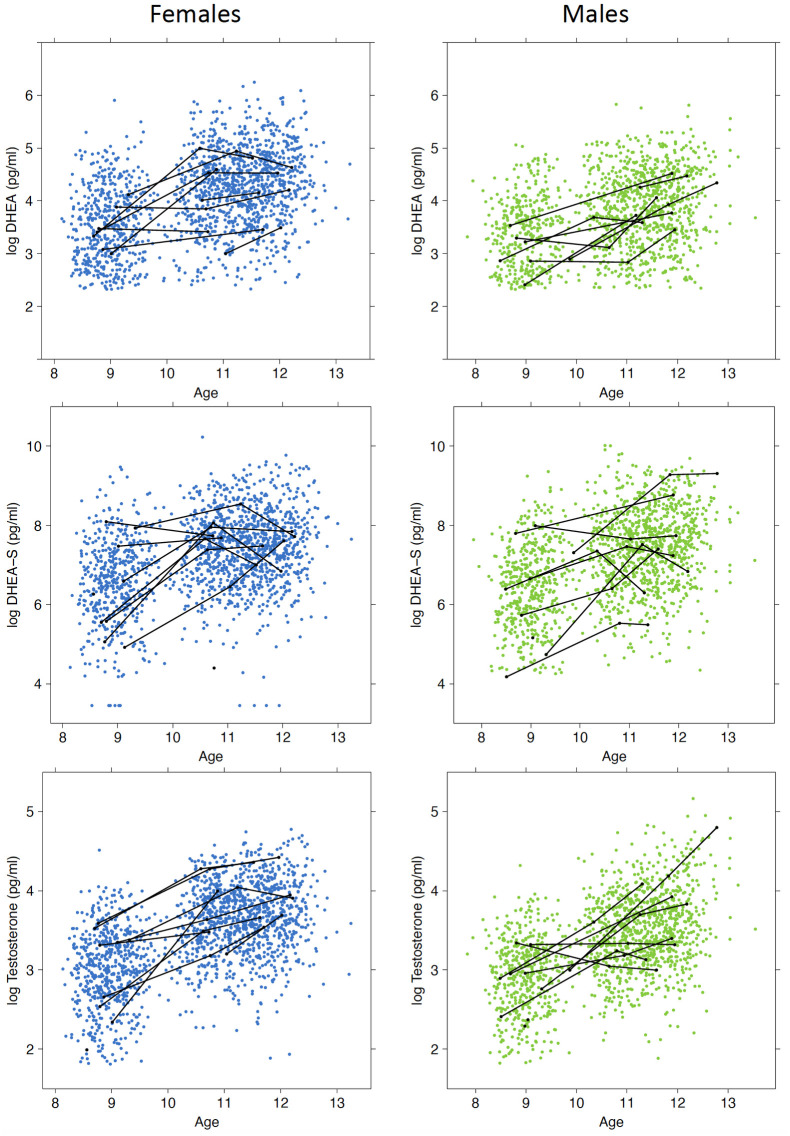
Scatterplots showing the relationship between age and (log-transformed) saliva hormone levels for females and males with hormone values above the detection limit. To illustrate the longitudinal nature of the data, repeated measures over the follow-up period have been connected (black lines) for 10 randomly selected individuals.

Descriptive statistics for participant age, timing of saliva collection, and salivary hormone concentration at waves 1 to 4 are shown in Table 1. Initially, 1,239 students (667 females) consented to participate in CATS. Overall, 47% (n = 579) of participants identified as Anglo-Celtic or European, 8% (n = 98) East Asian or Southeast Asian, 4% (n = 54) South Asian or Middle Eastern, and <2% from each of the following: Aboriginal and Torres Strait Islander, Sub-Saharan Africa, or other, and 5% (n = 60) identified with more than one group. Information on ethnic or cultural background was not available for 34% of the participants. The range of log-transformed hormone values can be seen in Fig 1. A higher proportion of values were below the detection limit for males than females, and wave 1 had the highest proportion of undetectable values (Table 1). Of all available measures, a higher proportion of DHEA values were below the detection limit (11.3%) than DHEA-S (1.2%) and testosterone (1.7%). For all hormones, >70% participants had measurements available across three waves of data collection (S2 Table).

### Linear mixed-effects models

Table 2 presents the fixed-effect coefficient estimates for adrenal hormones in the mixed-effects models. On average, all hormone levels increased with age and generally decreased with later saliva collection time, for both sexes. As expected, increased saliva flow rate was associated with decreased DHEA-S levels, more so in females than males. Fixed intercept estimates in Table 2 provide the estimated population average log hormone levels at 9am (and log flow rate 0 for DHEA-S) at age 9 years among participants.

**Table 2:** Estimates of fixed-effect coefficients, with 95% confidence intervals, in the mixed-effects models.

	Females			Males		
	DHEA	DHEA-S	Testosterone	DHEA	DHEA-S	Testosterone
Age	0.36 (0.30, 0.42)	0.33 (0.26, 0.40)	0.27 (0.23, 0.31)	0.26 (0.20, 0.31)	0.38 (0.32, 0.44)	0.25 (0.21, 0.30)
Timing	-0.04 (-0.15, 0.07)	-0.17 (-0.31, -0.03)	-0.01 (-0.09, 0.07)	-0.04 (-0.16, 0.07)	-0.17 (-0.31, -0.03)	-0.02 (-0.10, 0.05)
Age×timing	-0.02 (-0.08, 0.04)	0.06 (-0.01, 0.14)	-0.02 (-0.06, 0.02)	0.03 (-0.03, 0.08)	0.06 (0.00, 0.12)	0.00 (-0.04, 0.03)
Flow	-	-0.18 (-0.24, -0.11)	-	-	-0.11 (-0.18, -0.04)	
Fixed intercept at 9 years old	3.38 (3.25, 3.50)	6.57 (6.42, 6.73)	3.02 (2.93, 3.11)	3.11 (2.98, 3.24)	6.51 (6.35, 6.66)	2.91 (2.82, 3.00)

Estimates of SDs and correlation parameters in mixed-effects models for hormone measures at 9 years old together with calculated VPCs are shown in Table 3.

**Table 3:** Estimates of standard deviation and correlation parameters in the mixed-effects models for adrenal hormone measured at age 9 years old.

		Batch int SD	Individual int SD	slope SD	err SD	Slope-int Corr	VPC
**Females**	Log DHEA	0.18	0.66	0.14	0.43	-0.36	0.66
	Log DHEA-S	0.22	0.86	0.13	0.54	-0.47	0.68
	Log Testosterone	0.16	0.41	0.09	0.29	-0.48	0.61
**Males**	Log DHEA	0.17	0.60	0.13	0.49	-0.09	0.56
	Log DHEA-S	0.14	0.92	0.13	0.56	-0.36	0.72
	Log Testosterone	0.15	0.40	0.13	0.29	-0.51	0.59

Abbreviations: SD: standard deviation; int: intercept; Corr: correlation (between random slopes and random intercepts); err: random error; VPC: variance partitioning coefficient;

^a^Changing the centring of age does not affect the variance of the random batch effects, random slopes or error terms.

For all hormones, the between-individual variation in hormone levels at age nine (i.e., timing; the individual intercept SDs in Table 3) were moderately large compared to the population average hormone levels at 9 years old (indicated by the fixed intercepts shown in Table 2). Also, the between-individual variations in hormone progression over time (i.e., tempo; the slope SDs in Table 3) were of moderate magnitude compared to the population average slopes (age coefficient (Table 2)). However, the small population average slopes compared with average hormone levels at 9 years old means that the between-individual variation in progression rates was of less importance for modelling the hormone trajectories.

The correlations between random intercepts and slopes were negative for all three hormones in both sexes.

For all three hormones and both sexes, VPC measures were between 0.56 and 0.72, suggesting that within-individual and between-batch variation was less than between-individual variation. The higher the VPC, the less within-individual and between-batch variation, meaning hormone levels for an individual are more stable and measurements more reliable.

### Classes based on hormone levels at age 9 years old, rate of progression during follow-up period, and cross-classified trajectories

Classification of individuals by hormone level at age 9 years (timing) or hormone progression rate over time (tempo) are shown in Table 4.

**Table 4:** Number (%) of females and males in classes based on hormone levels (L1: Low-level, L2: Mid-level, or L3: High-level compared to population) at age 9 years old and rate of progression during follow-up period (R1: Slow/normal rate, R2: Fast rate compared to population).

		Females			Males		
		DHEA	DHEA-S	Testosterone	DHEA	DHEA-S	Testosterone
**Timing**	**L1**	207 (31.3)	193 (29.3)	183 (27.7)	190 (33.6)	173 (30.7)	164 (29.0)
	**L2**	246 (37.2)	245 (37.2)	262 (39.6)	191 (33.8)	191 (33.9)	227 (40.2)
	**L3**	208 (31.5)	221 (33.5)	216 (32.7)	185 (32.7)	199 (35.4)	174 (30.8)
**Tempo**	**R1**	336 (50.8)	335 (50.8)	346 (52.3)	280 (49.5)	284 (50.4)	299 (52.9)
	**R2**	325 (49.2)	324 (49.2)	315 (47.7)	286 (50.5)	279 (49.6)	266 (47.1)
Missing^a^		6	8	6	6	9	7

^a^ Classification missing due to missing measurements across all waves

For tempo, for all hormones, the pattern was similar for females and males, with almost equal proportions in the R1 and R2 classes.

For timing, the pattern was similar for females and males for testosterone, with the largest proportion of individuals in the L2 class (~40%) and similar proportions in the L1 or L3 classes (28% to 33%). Also, for DHEA and DHEA-S, the largest proportion of females were in the L2 class (37%). For males, similar proportions were in each of the L1, L2, and L3 classes, for DHEA and DHEA-S.

Cross-classification of individuals by timing (at age 9 years) and tempo are shown in Table 5. For DHEA-S and testosterone, the patterns were broadly similar in females and males. For DHEA-S, the largest proportion of individuals were in the L3/R1 class, meaning that they had relatively high DHEA-S levels at age 9, and relatively slower tempo (27% females, 26% males). This was followed by L1/R2; those with relatively low levels initially and a faster tempo (25% females, 22% males). For testosterone, the largest proportion of individuals were in the L3/R1 (24% females, 22% males) or L2/R1 (22% females, 23% males) classes. For DHEA, the pattern was different for females and males, with the largest proportion of females (23%) being in the L3/R1 class but the highest proportion of males (21%) in the L1/R1 class, representing those with relatively low DHEA levels and a relatively slower tempo.

**Table 5:** Number (%) of females and males in cross-classifications of individuals by hormone level at age 9 years and hormone progression rate over time. L1, L2, and L3 respectively indicate low, normal, and high predicted hormone levels compared to population; R1 and R2 indicate slow/normal and fast predicted progression rate over time compared to population.

			DHEA		DHEA-S		Testosterone	
	Progression rate over time		R1	R2	R1	R2	R1	R2
**Females**	**Level class at age 9**	**L1**	69 (10.4)	138 (20.9)	31 (4.7)	162 (24.6)	46 (7.0)	137 (20.7)
		**L2**	115 (17.4)	131 (19.8)	125 (19.0)	120 (18.2)	144 (21.8)	118 (17.9)
		**L3**	152 (23.0)	56 (8.5)	179 (27.2)	42 (6.4)	156 (23.6)	60 (9.1)
		Missing^a^	6		8		6	
**Males**		**L1**	120 (21.2)	70 (12.4)	47 (8.3)	126 (22.4)	49 (8.7)	115 (20.4)
		**L2**	82 (14.5)	109 (19.3)	92 (16.3)	99 (17.6)	127 (22.5)	100 (17.7)
		**L3**	78 (13.8)	107 (18.9)	145 (25.8)	54 (9.6)	123 (21.8)	51 (9.0)
		Missing^a^	6		9		7	

^a^Classification missing due to missing measurements across all waves.

**Fig 2:**
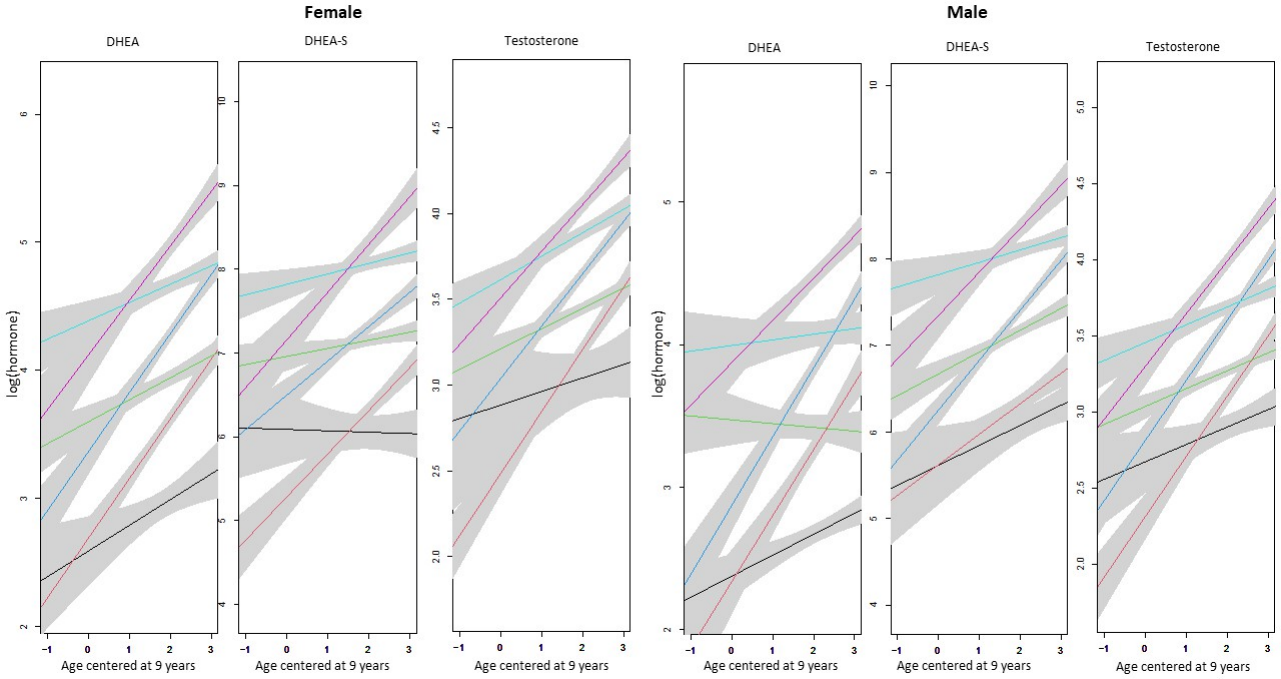
Plots of estimated mean trajectories within each class of individuals defined by hormone level at age 9 years (x axis is age centred at 9 years) and hormone progression rate over time with timing of collection at 9am (and log flowrate 0 for DHEA-S). The grey bands represent the 95% confidence interval around the estimated mean trajectories.

Fig 2 shows plots of mean trajectories within each cross-classification of individuals.

### Additional analyses

For all hormones and both sexes, the majority of participants (>96%) remained in the same class for timing and tempo after excluding saliva samples collected after 11am (n = 51) or samples not collected at school (n = 149) (S3 and S4 Tables).

## Discussion

To our knowledge this is the first study to use a longitudinal sample of children transitioning to adolescence with repeated salivary DHEA, DHEA-S and testosterone concentrations, and linear mixed-effects modelling techniques, to derive measures of adrenarcheal timing and tempo. For all hormones and both sexes, between-individual variation in hormone levels at age 9 (timing) were moderately large, while the size and variation observed in the tempo (i.e., slopes) was small, suggesting that variation in individual timing of adrenarche played a much greater role than tempo for determining an individual’s hormone trajectories relative to the population.

Our findings are in keeping with data published from the Growth and Obesity Chilean Cohort Study (n = 504 females) which showed that girls with relatively higher levels of DHEA-S at age 7 had persistently higher DHEA-S levels through late childhood until after menarche, higher testosterone levels until after thelarche, and was associated with earlier thelarche and menarche, although no differences in tempo of phenotypic puberty changes were found [9]. Together, our studies suggest that adrenal hormone levels during late childhood and early adolescence are predominantly driven by adrenarcheal timing, with tempo having limited impact. This finding is significant as it suggests that adrenal hormonal levels at age 7–9 years can be used to predict relative levels in later childhood (up to 12–13 years). Importantly, our analyses focussed on biochemical rather than phenotypic adrenarcheal changes, which do not necessarily correlate [20, 21], and further work would be needed to understand the variation in phenotypic adrenarche.

Our data represent a large population-based sample of repeated hormone measures of children aged 8–13 years and are subject to some limitations. While the majority of the saliva samples were collected according to the study protocol, a small number were collected later in the day or not in the school environment. Sensitivity analyses suggest that this variation did not significantly impact our results. These data were analysed using immunoassays [22]. While this technique allows analysis of large samples in a cost-effective manner, it is subject to limitations regarding specificity of analytes and accuracy. Future studies may consider alternative methodologies including mass spectrometry [23]. Overall, the quality of hormone measurements was reasonable in this study, but higher coefficients of variation in sample testing for DHEA, (intraassay CV 9.9%, interassay CV 16.5%), could suggest a degree of error in the DHEA measurements [24]. This may also reflect the generally low DHEA levels in younger participants in the study, which are close to the lower limit of detection for the assay, increasing potential inaccuracies. Cohort attrition is always a challenge for longitudinal studies. Our follow-up rates of >80% are high for a population-based study, and we used methods that rely on relaxed assumptions about missing data (compared to a complete case analysis), nevertheless these assumptions are unverifiable so loss to follow-up remains a limitation of our study.

While we collected three hormonal datapoints for this sample for each individual, our data collection started at approximately age 9 years meaning that we likely missed the earliest signs of adrenarche, which starts around 6 to 8 years of age [1] for many individuals. As we did not have data for younger participants to capture adrenarche initiation for all, we used level of hormones at age 8–9 years as a proxy for adrenarcheal timing, on the assumption that those participants who had experienced adrenarche at a younger age would be expressing higher adrenarcheal hormone levels by timepoint 1 than those who had only recently reached adrenarche. Future studies would need to include data from younger participants to test this assumption. By the later study timepoints, many participants will have experienced gonadarche which results in significant increased production of gonadal hormones, particularly testosterone in males. The salivary testosterone hormone data for our sample were in keeping with other published data for adolescent participants (see e.g., [25]) reflecting that although some of our participants have started gonadarche, their hormonal levels are still expected to be significantly below adult concentrations. This gonadal hormone production cannot be easily distinguished from adrenal production, and our analyses likely incorporate some gonadal-derived hormone and should be interpreted with this caveat. Nonetheless, we note that our findings about the trajectories for testosterone were similar to those for DHEA-S, which is predominantly produced in the adrenal glands even after gonadarche.

In addition, our model assumed that age, as well as time at saliva collection and age×time interaction had linear associations with hormone levels, implying that changes in hormone level changes were steady in relation to these variables, which is an over-simplification of this complex biological process. The simple linear trend was judged to be adequate to obtain interpretable summaries of change across the follow-up period, and more complex models (e.g. quadratic trends, spline modelling) were not appropriate given the number of longitudinal datapoints [5]. Additional, more frequent sampling across a wider age range would allow for the use of more complex statistical modelling, which could capture more of the inherent biological variation of the population.

## Conclusion

This paper described and classified adrenal trajectories, in terms of timing and tempo, in a population of children transitioning to adolescence with repeated measurements of salivary DHEA, DHEA-S, and testosterone. The mixed-effects modelling approach enabled meaningful partitioning of sources of variation in these longitudinal data and suggested that between-individual variation in timing was more important for determining relative adrenal hormonal level in childhood than tempo. Also, our findings suggest that adrenal hormonal level at single time point at 8–9 years old may be enough to predict relative levels in early adolescence (up to 13 years). The findings provide practical guidance for future studies attempting to measure adrenarche.

## Supporting information

S1 Table Specifications of Salimetrics salivary DHEA, DHEA-S, and testosterone assays. (PDF)

S2 Table Number (%) of waves with available hormone measurements for each hormone for females and males. (PDF)

S3 Table Percentage of individuals who changed class after excluding hormone measurements collected after 11am (n = 51). (PDF)

S4 Table Percentage of individuals who changed class after excluding hormone measurements not collected at school setting (n = 149). (PDF)

S1 Fig Histogram of log detectable values for DHEA-S overlaid with a normal distribution curve. (PDF)

S1 File Linear mixed-effect models for saliva hormones. (PDF)
