# Supplementary material for: Modelling timing and tempo of adrenarche in a prospective cohort study
Source: PLoS One. 2022 Dec 15;17(12):e0278948. doi: 10.1371/journal.pone.0278948 (PMC9754191; doi:10.1371/journal.pone.0278948)
Supplement: S1 Table — (PDF) [file pone.0278948.s001.pdf]

Modelling timing and tempo of adrenarche in a prospective cohort study

S. Ghazaleh Dashti, Lisa Mundy, Anne-Lise Goddings, Louise Canterford, Russell M. Viner, John B. Carlin, George Patton, Margarita Moreno-Betancur

Supporting information

S1 Table – Specifications of Salimetrics salivary DHEA, DHEA-S, and testosterone assays

S1 Table – Specifications of Salimetrics salivary DHEA, DHEA-S, and testosterone assays

	**DHEA**	**DHEA-S**	**Testosterone**
Analytical sensitivity [a]	5 pg/mL	95.14 pg/mL	<1.0 pg/ mL
Functional sensitivity [b]	8.32 pg/mL	266.61 pg/mL	0.67 pg/ mL
Assay Range	10.2 to 1000 pg/mL	62.9 to 15300 pg/mL [c]	6.1 to 600 pg/mL
Intra-assay CV	9.9%	8.3%	7.8%
Interassay CV	16.5%	9.1%	13.2%
Cross-reactivity	**Not detected**: 17β-Estradiol, Estriol, Estrone, Progesterone, 17α-Hydroxyprogesterone, Testosterone, Dihyroxytestosterone, Dianbol, 1s1-Hydroxytestosterone, 19-Nortestosterone, Cortisol, Aldosterone, Cortisone, 11-Deoxycortisol, 21-Deoxycortisol, Triamincinolone, Corticosterone, Transferrin,<br>**0.0378%**: Androstenedione<br>**0.063%**: DHEA-S	**Not detected**: Estradiol, Estriol, Progesterone, 17α-Hydroxyprogesterone, Testosterone, Cortisol, DHEA, Aldosterone, Cortisone, 11-Deoxycortisol, 21-Deoxycortisol, Dexamethasone, Triamincinolone, Corticosterone, Prednisolone, Prednisone, Transferrin, DHT, Dianbol, 19-Nortestosterone, 11-Hydroxytestosterone, Estrone<br>**0.0268%**: Transandrosterone<br>**0.0844%**: Androstenedione	**Not detected**: Aldosterone, Corticosterone, Cortisol, Cortisone, 11-Deoxycortisol, DHEA, 17α-Hydroxyprogesterone, Transferrin **0.004%**: 21-Deoxycortisol<br>**0.005%**: Estrone, Progesterone<br>**0.012%**: Estriol<br>**0.025%**: Estradiol<br>**0.165%**: Epitestosterone<br>**0.489%**: Dianabol<br>**1.157%**: Androstenedione<br>**1.90%**: 11-Hydroxytestosterone<br>**21.02%**: 19-Nortestosterone<br>**36.4%**: Dihydrotestosterone

Abbreviations: CV coefficient of variation

[a] Analytical sensitivity (limit of detection) defined as minimal hormone concentration that can be distinguished from 0

[b] Functional sensitivity (limit of quantitation) defined as concentration level resulting in a CV of 20% or less

[c] Following the manufacturer's instructions, samples with DHEA-S values greater than 15,300 pg/mL were diluted with DHEA-S diluent, retested, and the newly obtained values were multiplied by the dilution factor
