# Supplementary material for: Modelling timing and tempo of adrenarche in a prospective cohort study
Source: PLoS One. 2022 Dec 15;17(12):e0278948. doi: 10.1371/journal.pone.0278948 (PMC9754191; doi:10.1371/journal.pone.0278948)
Supplement: S2 Table — (PDF) [file pone.0278948.s002.pdf]

3Modelling timing and tempo of adrenarche in a prospective cohort study

S. Ghazaleh Dashti, Lisa Mundy, Anne-Lise Goddings, Louise Canterford, Russell M. Viner, John B. Carlin, George Patton, Margarita Moreno-Betancur

Supporting information

S4 Table – Number (%) of waves with available hormone measurements for each hormone for females and males

S4 Table – Number (%) of waves with available hormone measurements for each hormone for females and males

	Females (n=667)			Males (n=572)		
	DHEA	DHEA-S	Testosterone	DHEA	DHEA-S	Testosterone
3 waves	498 (74.6)	477 (71.5)	495 (74.2)	444 (77.6)	435 (76.0)	436 (76.2)
2 waves	95 (14.2)	110 (16.5)	98 (14.7)	68 (11.9)	72 (15.6)	73 (12.8)
1 wave	68 (10.2)	72 (10.8)	68 (10.2)	54 (9.4)	56 (9.8)	56 (9.8)
No wave	6 (0.9)	8 (1.2)	6 (0.9)	6 (1.0)	9 (1.6)	7 (1.2)
Total number of hormone values available for each analysis	1752	1723	1749	1522	1505	1510
