# Supplementary material for: Modelling timing and tempo of adrenarche in a prospective cohort study
Source: PLoS One. 2022 Dec 15;17(12):e0278948. doi: 10.1371/journal.pone.0278948 (PMC9754191; doi:10.1371/journal.pone.0278948)
Supplement: S3 Table — (PDF) [file pone.0278948.s003.pdf]

Modelling timing and tempo of adrenarche in a prospective cohort study

S. Ghazaleh Dashti, Lisa Mundy, Anne-Lise Goddings, Louise Canterford, Russell M. Viner, John B. Carlin, George Patton, Margarita Moreno-Betancur

Supporting information

S5 Table – Percentage of individuals who changed class after excluding hormone measurements collected after 11am (n=51)

S5 Table – Percentage of individuals who changed class after excluding hormone measurements collected after 11am (n=51)

	Females			Males		
% who change class for:	DHEA	DHEA-S	Testosterone	DHEA	DHEA-S	Testosterone
hormone level at age 9	2.4	1.8	2.4	3.2	2.0	3.4
hormone progression rate over time	2.1	2.3	2.4	3.7	3.7	1.4
