# Supplementary material for: Modelling timing and tempo of adrenarche in a prospective cohort study
Source: PLoS One. 2022 Dec 15;17(12):e0278948. doi: 10.1371/journal.pone.0278948 (PMC9754191; doi:10.1371/journal.pone.0278948)
Supplement: S4 Table — (PDF) [file pone.0278948.s004.pdf]

Modelling timing and tempo of adrenarche in a prospective cohort study

S. Ghazaleh Dashti, Lisa Mundy, Anne-Lise Goddings, Louise Canterford, Russell M. Viner, John B. Carlin, George Patton, Margarita Moreno-Betancur

Supporting information

S6 Table – Percentage of individuals who changed class after excluding hormone measurements not collected at school setting (n=149)

S6 Table – Percentage of individuals who changed class after excluding hormone measurements not collected at school setting (n=149)

	Females			Males		
% who change class for:	DHEA	DHEA-S	Testosterone	DHEA	DHEA-S	Testosterone
hormone level at age 9	2.0	3.6	2.6	3.5	2.1	3.0
hormone progression rate over time	3.3	3.6	2.6	5.5	4.3	4.4
