# Supplementary material for: Modelling timing and tempo of adrenarche in a prospective cohort study
Source: PLoS One. 2022 Dec 15;17(12):e0278948. doi: 10.1371/journal.pone.0278948 (PMC9754191; doi:10.1371/journal.pone.0278948)

Modelling timing and tempo of adrenarche in a prospective cohort study

S. Ghazaleh Dashti, Lisa Mundy, Anne-Lise Goddings, Louise Canterford, Russell M. Viner, John B. Carlin, George Patton, Margarita Moreno-Betancur

Supporting information

S2 Figure – Histogram of log detectable values for DHEA-S overlaid with a normal distribution curve

S2 Figure - Histogram of log detectable values for DHEA-S overlaid with a normal distribution curve; red line represents the log detection limit and the blue line the value that divided the area below the detection limit by two; values below the detection limit (log(62.9)), depicted by the red line) were imputed by log(49.6).

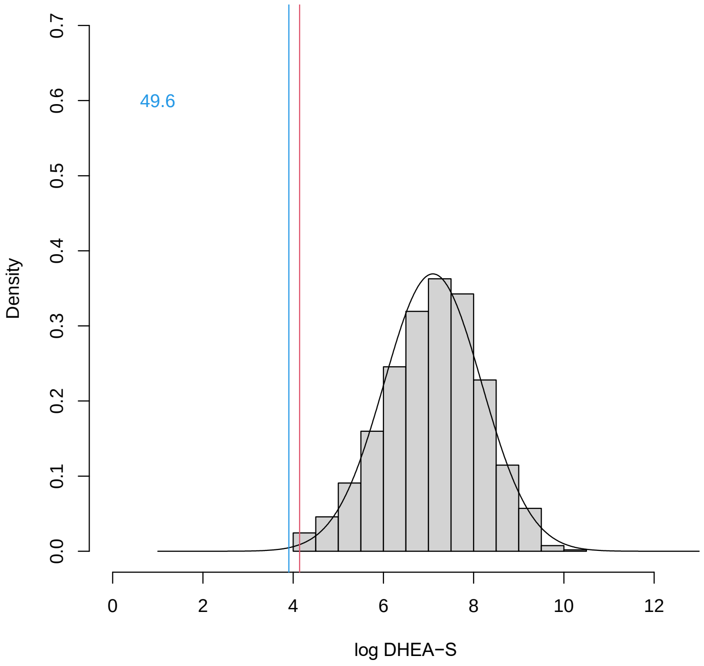

Supplement: S1 Fig — (PDF) [file pone.0278948.s005.pdf]
