# Supplementary material for: Modelling timing and tempo of adrenarche in a prospective cohort study
Source: PLoS One. 2022 Dec 15;17(12):e0278948. doi: 10.1371/journal.pone.0278948 (PMC9754191; doi:10.1371/journal.pone.0278948)
Supplement: S1 File — (PDF) [file pone.0278948.s006.pdf]

Modelling timing and tempo of adrenarche in a prospective cohort study

S. Ghazaleh Dashti, Lisa Mundy, Anne-Lise Goddings, Louise Canterford, Russell M. Viner, John B. Carlin, George Patton, Margarita Moreno-Betancur

Supporting information S3

Linear mixed-effect models for saliva hormones

For each hormone, linear mixed-effects models were fitted to the longitudinal hormone measurements including subject-specific random effects (intercept and age-slope) that represent individual measures of deviation from population trajectories. Models also included a batch-specific random effect to correct for batch effects. Fixed effects were specified for age centred at 9 years old, time at saliva collection centred at 9am (henceforth referred to simply as time), and an interaction between age and time. Specifically, hormone levels were modelled as increasing linearly with age and with time, with inclusion of an age×time interaction enabling the effect of diurnal variation to change as age increased. For (log) DHEA-S, the log-transformed flow rate was additionally included in all models with a fixed effect, because it is expected that the DHEA-S concentration (pg/ml) will fall as salivary flow (ml/min) increases [1]. Models were fitted using the standard method of restricted maximum likelihood [2].

To illustrate, below is the full linear mixed-effects model fitted to the log-transformed DHEA-S measures (denoted by Y_{ijk} for individual i, batch j, and measurement k), with fixed linear effects for centred age, centred time, age×time and log flow rate, as well as subject-specific random intercepts and age-slopes and batch-specific random intercepts:

$$Y_{ijk} = \beta_0 + \beta_1 a_{ijk} + \beta_2 t_{ijk} + \beta_3 a_{ijk} \times t_{ijk} + \beta_4 f_{ijk} + b_{0i} + b_{1i} a_{ijk} + b_{2j} + e_{ijk}$$

$$\text{where } a_{ijk} = \text{age}_{ijk} - 9;\ t_{ijk} = \text{time}_{ijk} - 9;\ f_{ijk} = \text{log flow rate}_{ijk}$$

The first five parameters in this model are the fixed-effect components and interpreted as follows: β_0 is the average log DHEA-S level for 9 year-olds at 9am and log flow rate 0; β_1 is the average change in log DHEA-S level with 1 year increment in age (with time at 9am and log flow rate fixed); β_2 is the average change in log DHEA-S level with 1 hour increment in time of saliva collection (with age at 9 years old and log flow rate fixed); β_3 the average difference in change in log DHEA-S level with 1 hour increment in time of saliva collection for 1 year increment in age (with log flow rate fixed); and β_4 is the average change in log DHEA-S level with 1 unit increment in log flow rate (with age and time fixed).

For individual i, b_{0i} is the subject-specific random intercept and provides a measure of deviation of individual i's hormone levels from the population average at 9 years old (with time and log flow rate fixed), and b_{1i} is the subject-specific random age-slope, and provides a measure of deviation of individual i's progression rate from the population average rate over the follow-up period (with time and log flow rate fixed). The batch-specific random intercept, b_{2j}, measures the deviation of batch j hormone levels from the population average at 9 years old, with time and log flow rate fixed. And e_{ijk} is a random error term. The model assumes that b_{0i}, b_{1i}, b_{2j}, and e_{ijk} follow normal distributions with mean 0 and variances $\sigma_{b_0}^2$, $\sigma_{b_1}^2$, $\sigma_{b_2}^2$ and σ_e^2 respectively.

Estimates of these variances can be used to estimate the variance partitioning coefficient (VPC), using the following formula:

$$\text{VPC(a)} = \frac{\sigma_{b_0}^2 + 2\sigma_{b_{01}} a_k + \sigma_{b_1}^2 a_k{}^2}{\sigma_{b_0}^2 + 2\sigma_{b_{01}} a_k + \sigma_{b_1}^2 a_k{}^2 + \sigma_{b_2}^2 + \sigma_e^2} \qquad 3$$

where $\sigma_{b_{01}}$ is the covariance between intercepts and slopes. In the absence of a random slope, the VPC would be equivalent to the standard intra-cluster correlation (ICC). It quantifies the proportion of the total variance in hormone measurements due to between-individual

variation (versus within-individual and between-batch variation) among measurements at a given age. The higher the VPC, the less within-individual and between-batch variation at each given age, meaning hormone levels for an individual are more stable and it is easier to obtain meaningful and reliable measures of these.

References

1. Vining RF, McGinley RA. The measurement of hormones in saliva: possibilities and pitfalls. *J Steroid Biochem*. 1987;27(1-3):81-94.
2. Laird NM, Ware JH. Random-effects models for longitudinal data. *Biometrics*. 1982;38(4):963-974.
3. Goldstein H, Browne W, Rasbash J. Partitioning Variation in Multilevel Models. *Understanding Statistics*. 2002;1(4):223.
